# Self-Sacrifice Is Not the Only Way to Practice Filial Piety for Chinese Adolescents in Conflict With Their Parents

**DOI:** 10.3389/fpsyg.2021.661335

**Published:** 2021-05-14

**Authors:** Chih-Wen Wu, Kuang-Hui Yeh

**Affiliations:** ^1^Department of Human Development & Family Studies, National Taiwan Normal University, Taipei, Taiwan; ^2^Institute of Ethnology, Academia Sinica, Taipei, Taiwan; ^3^Department of Psychology, National Taiwan University, Taipei, Taiwan

**Keywords:** filial piety, function-oriented appraisal of conflict, interpersonal conflict resolution strategy, parent–adolescent conflict, dual filial piety model

## Abstract

We applied the theoretical perspective of the dual filial piety model to consider the diversity of parent–child conflict resolution strategies in order to determine whether Chinese adolescents use strategies other than self-sacrifice to practice filial piety when in conflict with their parents. Study 1 utilized a cross-sectional design with 247 valid responses. The structural equation modeling analysis indicated that Taiwanese adolescents’ authoritarian filial piety (AFP) beliefs are positively related to use of a self-sacrifice strategy, and reciprocal filial piety (RFP) beliefs are positively related to use of compatibility and compromise strategies. Adolescents’ AFP and RFP beliefs are negatively related to use of utility and escape strategies. Study 2 applied a temporal separation procedure with a 1-year lag to remedy common method variance bias. Analysis of 1,063 valid responses replicated the findings of Study 1 and indicated that adolescents’ function-oriented appraisal of conflict can play a mediating role between RFP and the use of the compatibility and compromise strategies. These findings broaden the understanding of filial piety in modern Chinese societies and have implications for adolescents’ well-being and family life.

## Introduction

Conflict with parents is quite common in adolescence (Laursen and Collins, 2009). Researchers have focused on the different strategies adolescents tend to use to resolve conflict with parents (e.g., Van Doorn et al., 2008), and on the antecedent factors such as parental behavior (e.g., Merolla and Kam, 2017) that could influence an adolescent’s decision to adopt a particular strategy. For Chinese families, filial piety (*xiao*), the core virtue of Confucian philosophy, advocates that children should pay attention to their relationship with their parents and fulfill their parents’ expectations, even when conflict arises (Ho, 1994; Chen et al., 2007). This perspective on filial piety has resulted in the common perception that Chinese adolescents in conflict with their parents ought to practice filial piety by sacrificing their own volition.

However, since filial piety is a complex set of constructs with diverse aspects (Yeh, 2003; Yeh and Bedford, 2003), we expect that a variety of resolution strategies may be available for Chinese adolescents to practice filial piety and resolve conflict with their parents (Yeh and Bedford, 2004; Wu et al., 2019). The purpose of this article is to investigate whether Chinese adolescents can use conflict resolution strategies other than self-sacrifice to practice filial piety. We examined the effect of adolescents’ filial piety beliefs on their use of different conflict resolution strategies with two empirical studies. Study 1 used a cross-sectional design and Study 2 used a follow-up design with a 1-year lag. We also examined adolescents’ function-oriented appraisal of conflict as a potential mediator between filial belief and conflict resolution strategy.

### Filial Piety Beliefs

Researchers have proposed many different interpretations of filial piety. For example, Ho (1994) claimed that filial piety is a principle for parent–child interaction related to authoritarian moralism and can be an obstacle to the individual’s volition. Empirical findings have shown that filial belief is positively related to obedience to parental control, a neurotic disposition, and cognitive conservatism (Chen et al., 2007).

As another perspective, the dual filial piety model (DFPM; Yeh, 2003; Yeh and Bedford, 2003) suggests that filial piety encompasses two distinct aspects, namely reciprocal and authoritarian, which correspond to different Confucian ethical principles. Reciprocal filial piety (RFP) represents an aspect of parent–child interaction grounded in the interdependent affection and genuine gratitude of children toward their parents who made an effort to raise and support them. RFP reflects the intimacy principle (*qin-qin*) in Confucian philosophy, which requires that one favor those closest to oneself, as well as the reciprocity principle (*bao*), which suggests that one should be grateful and repay any kindness one receives. RFP reflects a basic need for relatedness and emotional safety between two individuals.

Previous research has found that RFP contributes to intimate parent–child relationships (Chen et al., 2016), fewer conflicts with parents (Li et al., 2014), a higher level of perspective taking and self-disclosure (Yeh and Bedford, 2003), and mature development of autonomy in terms of maintaining intergenerational harmony and expressing inner attributes (Yeh, 2014). RFP is also positively related to several personal adaptation indices, such as subjective happiness (Chen et al., 2016), life satisfaction (Leung et al., 2010), and mental health (Jen et al., 2019).

In contrast, authoritarian filial piety (AFP) represents another aspect of parent–child interaction based on the hierarchy in the family. Parents are the superordinate figures with legitimacy to govern, discipline, and even punish children. AFP reflects the respect principle (*zun-zun*) in Confucian philosophy, which states that one should submit to parental commands, expectations, and criticism. Because the contents of AFP have been deeply ingrained as a social constraint, obedience to parents is applied as a public standard for judging a person’s morality as a “good child/person” (Kwan, 2000).

Empirical findings have indicated that AFP is related to perceived higher parental control (Li et al., 2014; Yeh, 2014), a higher level of particularism, authoritarian aggression, and conventionalism (Yeh and Bedford, 2003), and stronger belief in the legitimacy of parental authority (Liu, 2013). Because AFP focuses on the hierarchical parent–child relationship, it provides a reason for children to worry about offending parental authority, and may result in children’s obeying parental requirements despite a loss of personal volition. AFP is associated with some maladaptation indices corresponding to intrapersonal stress, such as lower self-esteem (Leung et al., 2010) and higher level of depression, anxiety, aggression, and deviant behavior (Yeh, 2006).

These empirical studies of the DFPM have broadened the understanding of filial piety with its comprehensive influence on modern Chinese family life, both positive and negative. However, few researchers have investigated the role of RFP and AFP beliefs in parent–adolescent conflict or the resolution strategies corresponding to each of these two distinct aspects. Although AFP would seem to be related to self-sacrifice, the role of RFP is difficult to hypothesize. Does RFP also support adolescents in sacrificing their own volition to fulfill parental expectations? What strategies may be available for Chinese adolescents to achieve their personal volition and still practice filial piety?

### The Conflict Resolution Strategies

In Chinese society, the myth that self-sacrifice is the only way to practice filial piety largely results from the presupposition that parental expectations and personal goals are as incompatible as two ends of a single dimension. However, these two concerns could be better viewed as two independent dimensions that give rise to more than one strategy to fulfill parental expectations (Hammock et al., 1990). For example, Yeh (1995) combined two independent dimensions, *achieve personal goals* and *fulfill parental expectations*, to propose a five-strategy model: The *compatibility* strategy leads to a win-win resolution in which children fulfill parental expectations and simultaneously achieve their own goals; neither side need yield their goals and expectations. The *self-sacrifice* strategy results in an obedience resolution in which the children carry out parental expectations as the top priority and demonstrate their submission to parents by giving up their own goals. In contrast, the *utility* strategy results in a resolution in which the children ignore parental expectations to pursue their own goals. The *escape* strategy results in a resolution in which children are passive with regard to conflict with their parents, and so have no ideas or motivation for either their own goals or parental expectations. The *compromise* strategy leads to a resolution in which children attempt to achieve a middle ground wherein both sides must make some concessions.

In Yeh’s framework, the compatibility and compromise strategies are similar to the self-sacrifice strategy because all three involve a concern for parental expectations. This means that both the compatibility and compromise strategies could be alternative ways to practice filial piety and resolve conflict with their parents. Furthermore, these two strategies are better than self-sacrifice because they not only consider parental expectations but also target personal goals. If a child adopts either of these two strategies, s/he can still practice filial piety without totally sacrificing personal goals. Research has demonstrated that these two strategies are better than the self-sacrifice, utility, and escape strategies in that they have a greater association with a high quality parent–child relationship, a low frequency of parent–child conflict, and better personal adaptation (i.e., greater life satisfaction and fewer psychological symptoms, such as depression, anxiety, and stress) (Yeh and Tsao, 2014; Wu et al., 2019).

Previous researchers have investigated the relationship between the dual aspects of filial piety and these parent–child conflict resolution strategies. Yeh and Bedford (2004) found that Chinese adolescents endorsing higher RFP are more likely to adopt compatibility and compromise strategies, but less inclined toward the escape strategy. Those endorsing higher AFP tend to adopt a self-sacrifice strategy, and are less likely to apply the utility strategy. However, two limitations in this work are noteworthy. First, the measurement of these resolution strategies may have been unreliable because each was assessed with only two items, and the reliability coefficients were absent in the article. In addition, the authors did not specify the underlying mechanism linking filial piety belief to these resolution strategies. To overcome these limitations, we investigated the relation of RFP and AFP to these different strategies using measurement tools with acceptable reliability, and focused on adolescents’ appraisal of conflict as the potential mediator to explore the underlying mechanism.

### Conflict Resolution Strategies and Their Connections to Filial Beliefs

We suggest that adolescents who endorse AFP beliefs tend to interpret parental expectations in terms of the requirement to obey parental authority. With AFP, personal goals are seen as obstructions, which implies that adolescents’ personal goals and parental expectations are mutually exclusive or in competition with one another. In order to meet their basic need to conform to a “good child” identity, adolescents endorsing high AFP tend to adopt the self-sacrifice strategy and demonstrate unconditional submission (Yeh and Bedford, 2004).

However, adolescents who endorse RFP beliefs care about their parents’ expectations and the reasons those expectations are important to their parents. They do not just see conflict with their parents as obstacles to personal goals. The basic needs for relatedness and emotional safety, to which RFP corresponds, can coexist with the basic need for individuation without social constraint (Yeh and Yang, 2006). As RFP is positively associated with malleable thinking, such as perspective taking (Yeh and Bedford, 2003) and cognitive flexibility (Jen et al., 2019), it may broaden one’s mindset allowing conflict to be regarded as an opportunity in which one can not only learn more about what one’s parents expect, but also share their own goals that they want to pursue. Thus, adolescents endorsing high RFP tend to adopt compatibility and compromise strategies for meeting parental expectations and simultaneously pursuing personal goals.

### Appraisal of Parent–Child Conflict as a Mediator

As cognitive appraisal has a crucial role in the resolution of interpersonal conflict (Murray et al., 2006; Yeh, 2012), we propose cognitive appraisal of parent–child conflict as a potential mediator to elaborate the mechanism between RFP and adolescents’ use of compatibility and compromise strategies. RFP may contribute to a function-oriented appraisal of conflict (FAC), a concept Yeh (2012) proposed to reflect the belief that interpersonal conflict can be functional without necessarily requiring competition. Individuals adopting FAC believe that conflict can be an opportunity to foster mutual understanding, effectively eliminate a difference in opinion, and improve skill in parent–child interaction.

Existing research has demonstrated the tendency to adopt compatibility and compromise strategies (but not utility or escape strategies) is positively associated with FAC for parent–child relationships (Yeh and Tsao, 2014) and romantic relationships (Chiao et al., 2018). We thus believe that RFP may relate to the recognition that personal goals and parental expectations can be achieved simultaneously through FAC, which would allow adolescents’ use of compatibility and compromise strategies to resolve conflict with parents.

### Overview

In this study, we first hypothesize that adolescent endorsement of AFP beliefs has a positive correlation with use of the self-sacrifice strategy when in conflict with their parents (Hypothesis 1), and that adolescent endorsement of RFP beliefs has a positive correlation with use of the compatibility and compromise strategies (Hypothesis 2). We also hypothesize that greater adolescent endorsement of both RFP and AFP beliefs, will correspond to reduced use of the utility and escape strategies (Hypothesis 3). We expect FAC to be a mediator between RFP belief and the tendency to adopt compatibility and compromise strategies (Hypothesis 4).

In accordance with previous research, fathers in Chinese families usually enact the authority role to maintain the hierarchy of the family, while mothers assume the role of caregiver to connect affection among family members (e.g., Shek et al., 2000; Ho et al., 2010). Considering that the role difference between fathers and mothers may confound our research findings, we asked participants to consider the separate contexts of father–child and mother–child interaction.

We conducted two empirical studies, Study 1 applied a cross-sectional design to test Hypotheses 1, 2, and 3, and Study 2 adopted a follow-up design with a 1-year lag to replicate the findings from Study 1 and tested Hypothesis 4.

## Study 1

### Participants and Procedures

A total of 253 high school students were recruited as participants. After gaining their and their parents’ informed consent, participants answered the father–child and mother–child versions of the questionnaires in counterbalanced order. The total valid sample size was 247 (93 females), with 222 completing both versions of the questionnaire, and 9 and 16 completing only the father– or mother–child versions, respectively. The valid sample size was 231 and 238 for the father–child and mother–child versions, respectively. Participants’ mean age was 16.11 years (*SD* = 0.39). The mean ages of the fathers and mothers were 47.59 years (*SD* = 5.60) and 44.24 years (*SD* = 5.00), respectively. The percentages of fathers and mothers with each education level was as follows: junior high school or below, 17% and 15%; senior high school, 50% and 51%; college, graduate school, or above, 34% for both.

### Measures

#### Filial Piety Belief

We adopted the dual filial piety scale (Yeh and Bedford, 2003) and adjusted the parental term for father–child and mother–child versions. The RFP subscale has eight items to measure participants’ beliefs that children ought to provide emotional support for and take authentic care of their father/mother (e.g., “Support my father’s/mother’s livelihood to make his/her life more comfortable”). The AFP subscale has eight items that measure participants’ beliefs that children ought to respect the hierarchical relationship between parents and children (e.g., “Do whatever my father/mother asks me to do right away”). Participants responded on a six-point Likert scale ranging from 1 (*Extremely unimportant*) to 6 (*Extremely important*). The Cronbach’s α of the RFP subscale was 0.95 and 0.96 for father–child and mother–child versions, and that of AFP was 0.89 for both versions.

#### Parent–Adolescent Conflict Resolution Strategy

We used the parent–adolescent conflict resolution strategy scale (Wu et al., 2019), which comprises five strategies: compatibility (e.g., I try my best to work with my father/mother to reach a consensus with which both parties are satisfied), compromise (e.g., I deal with the conflict with father/mother through a way that meets each other halfway), self-sacrifice (e.g., I give up my interest, giving priority to my father’s/mother’s request), utility (e.g., I stick to my opinion until my father/mother is willing to accept my claim), and escape (e.g., I leave the conflict with my father/mother alone, pretending it never happened). Each strategy contains four items with a five-point Likert scale ranging from 1 (*Never*) to 5 (*Always*). The Cronbach’s α of each subscale for father–child version were 0.86, 0.79, 0.77, 0.80, and 0.76, and those for mother–child version were 0.88, 0.79, 0.81, 0.78, and 0.75, respectively.

### Analysis Strategy

We used Mplus (version 8) to calculate the descriptive statistics of the major variables and to test our hypothetical structural equation models. We adopted the item-to-construct balancing procedure (Little et al., 2002) to parcel out the RFP and AFP items into three indicators to simplify the measurement models. We did not parcel the subscales of resolution strategies as each has only four items, and thus would not contribute to simplification.

We used the ratio of χ^2^ to *df* (χ^2^*/df*), the comparative fit index (CFI), Tucker–Lewis index (TLI), root mean square error of approximation (RMSEA), and standardized root mean square residual (SRMR) to test the model-fit. The model fit is considered acceptable when χ^2^*/df* is lower than 5 (Schumacker and Lomax, 2004), CFI and TLI are higher than 0.90, and RMSEA and SRMR are lower than 0.080 (Hu and Bentler, 1999; McDonald and Ho, 2002). We computed the influence of participants’ gender as a control variable because both the male and female sample sizes were too small to examine the effect of participants’ gender. We also computed the influence of parental education level as another control variable (1 = junior high school or below; 2 = senior high school; 3 = college, graduate school, or above).

### Results and Discussion

The means, standard deviations, and correlation coefficients of the major variables are summarized in Table 1. For the father–child and mother–child datasets, both RFP and AFP had significantly positive correlations with compatibility, compromise, and self-sacrifice strategies, but a negative correlation with the utility and escape strategies.

**TABLE 1:** Correlations, means, and standard deviations of the main variables in Study 1.

	***Correlation coefficients***								
	***1***	***2***	***3***	***4***	***5***	***6***	***7***	***M***	***SD***
1. Reciprocal filial piety		0.46**	0.45**	0.37**	0.14*	−0.25**	−0.31**	4.95	1.06
2. Authoritarian filial piety	0.50**		0.31**	0.27**	0.39**	−0.23**	−0.27**	3.25	1.08
3. Compatibility strategy	0.43**	0.35**		0.79**	0.56**	0.01	−0.22**	2.74	1.04
4. Compromise strategy	0.40**	0.34**	0.78**		0.61**	0.18**	−0.07	2.54	0.91
5. Self-sacrifice strategy	0.23**	0.52**	0.42**	0.50**		0.16*	0.07	2.07	0.85
6. Utility strategy	−0.28**	−0.26**	0.01	0.06	−0.10		0.44**	2.45	0.93
7. Escape strategy	−0.30**	−0.26**	−0.18**	−0.09	0.05	0.46**		2.36	0.97
*M*	4.82	3.14	2.49	2.28	1.90	2.35	2.42		
*SD*	1.04	1.05	1.03	0.85	0.77	0.98	1.00		

Numbers below the diagonal are from the father–child dataset (*n* = 231); those above are from the mother–child dataset (*n* = 238).

*p < 0.05; **p < 0.01.

**FIGURE 1:**
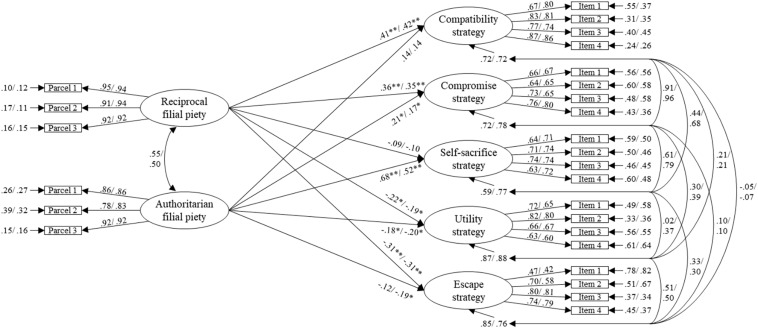
Structural model of dual filial piety and parent–adolescent conflict resolution strategy in Study 1. The influence of the adolescent’s gender has been controlled in this model. All coefficients are standardized solutions; the first coefficient is the father–child model (*n* = 231), the second is the mother–child model (*n* = 238). *p < 0.05, **p < 0.01.

We investigated the effect of filial piety beliefs on different resolution strategies with the hypothetical model presented in Figure 1. For the father–child model, the model-fit was acceptable with χ^2^(316, *N* = 231) = 589.58, *p* < 0.001, χ^2^/*df* = 1.87, CFI = 0.92, TLI = 0.90, RMSEA = 0.061, SRMR = 0.055, and all loadings were significant (*p*s < 0.01). For the mother–child model, the model-fit was also acceptable with χ^2^(316, *N* = 238) = 592.62, *p* < 0.001, χ^2^/*df* = 1.88, CFI = 0.92, TLI = 0.91, RMSEA = 0.061, SRMR = 0.057, and all loadings were significant (*p*s < 0.001).

For the compatibility strategy, the effect of RFP for both father–child (γ = 0.41, SE = 0.07) and mother–child models (γ = 0.42, SE = 0.07) was positively significant (*p*s < 0.001), but that of AFP for both models was not significant. For the compromise strategy, RFP’s effect for both father–child (γ = 0.36, SE = 0.08) and mother–child models (γ = 0.35, SE = 0.08) was positively significant (*p*s < 0.001). AFP’s effect was smaller, but it was still positively significant for both father–child (γ = 0.21, SE = 0.08, *p* = 0.01) and mother–child models (γ = 0.17, SE = 0.08, *p* = 0.04). For self-sacrifice strategy, although RFP’s effect was not significant, AFP’s for both models (father–child: γ = 0.68, SE = 0.07; mother–child: γ = 0.52, SE = 0.07) was positively significant (*p*s < 0.001). For the utility strategy, RFP’s effect was negatively significant for both models (father–child γ = −0.22, SE = 0.09, *p* = 0.01; mother–child γ = −0.19, SE = 0.08, *p* = 0.02), and so was AFP’s effect (γ = −0.18, SE = 0.09, *p* = 0.04; γ = −0.20, SE = 0.09, *p* = 0.02, respectively). For the escape strategy, RFP’s effect for both models (father–child: γ = −0.31, SE = 0.09; mother–child: γ = −0.31, SE = 0.08) was negatively significant (*p*s < 0.01), but AFP’s effect was negatively significant only for the mother–child model (γ = −0.19, SE = 0.08, *p* = 0.02), and not for the father–child model. These findings support Hypotheses 1 and 2. Our results verified the common perception that adolescents’ tendency to sacrifice personal goals to fulfill parental expectations is associated with AFP beliefs. However, adolescents who endorsed RFP beliefs indicated use of compatibility and compromise strategies to meet filial requirements while pursuing personal goals. Hypothesis 3 was partially supported in that both RFP and AFP were negatively related to the strategies entailing a lack of concern for parental expectations, namely utility and escape, except for the relationship between AFP and escape strategy for the father–child model. We discuss this exception in the final section.

Our results also indicated that AFP is related to the compromise strategy, which may be part of a compound strategy entailing use of the self-sacrifice and compromise strategies together. Adolescents who endorse AFP beliefs may first adopt the self-sacrifice strategy as a probe. If their parents respond with concessions, they may switch to the compromise strategy and propose some part of their personal goals.

Three limitations we further considered and tried to deal with in Study 2. First, to further elaborate the mechanism linking RFP and the tendency to use the compatibility and compromise strategies, Study 2 investigated adolescents’ FAC as a mediator, which reflects the belief that interpersonal conflicts can facilitate mutual understanding and problem solving (Yeh, 2012). FAC is positively associated with the use of compatibility and compromise strategies to resolve interpersonal conflict (Yeh and Tsao, 2014; Chiao et al., 2018). In addition, common method variance bias could be confounding the findings from Study 1 due to the cross-sectional design. To remedy this bias, we adopted a follow-up design with temporal separation procedure suggested by Podsakoff et al. (2003) in Study 2. Last, previous research pointed out that daughters in Chinese families pay more attention to having an intimate connection with parents than sons do (e.g., Shek, 2000; Chao, 2008), so we further investigated whether gender differences exist by recruiting a sufficient sample size in Study 2.

## Study 2

### Participants and Procedures

We recruited Taiwanese adolescents who did not participate in Study 1. In this Study, participants have to complete the measurements of predictor, mediator, and criteria variables at two time points. At these two time points, we obtained informed consent from them and their parents. The questionnaire at Time 1 measured participants’ filial piety beliefs and FAC, while that at Time 2 measured their tendency to adopt different conflict resolution strategies. Participants completed the father–child and mother–child versions in counterbalanced order. The sample size was 1,174 at Time 1 and 1,096 at Time 2 after a 1-year lag. Thirty-three samples that had in-completed responses at Time 2 were excluded as well. The total valid sample completing the questionnaires at both time points was 1,063 (638 female), with 898 answering both versions, and 46 and 119 only answering the father– or mother–child version, respectively. We obtained, 944 and 1,017 valid responses for the father– and mother–child versions, respectively. Participants had a mean age of 16.05 years (*SD* = 0.39) at Time 1. The mean ages of their fathers and mothers at Time 1 were 47.76 years (*SD* = 5.24) and 44.74 years (*SD* = 5.05), respectively. The percentage of fathers and mothers at each education level was as follows: junior high school or below, 18% and 17%; senior high school, 49% and 53%; and college, graduate school, or above, 33% and 30%, respectively.

### Measures

#### Filial Piety Belief

We used the same scale as in Study 1. The Cronbach’s αs for the father–child and mother–child versions of the RFP subscales were 0.94 and 0.93, respectively. For the AFP subscales, they were 0.85 and 0.83.

#### Function-Oriented Appraisal

We derived the function-oriented appraisal of conflict scale from Yeh (2012) model of the constructive transformation process. This scale contains 10 items to measure participants’ agreement with some adaptive values when in conflict with their parents, such as the opportunity to foster mutual understanding, reduce discrepancy, and improve skill in parent–child interaction. The items are measured with a five-point Likert-type scale ranging from 1 (*Not agree at all*) to 5 (*Always agree*). The Cronbach’s α was 0.93 for both father–child and mother–child versions.

#### Parent–Adolescent Conflict Resolution Strategy

We used the same scale as in Study 1. The Cronbach’s α of compatibility, compromise, self-sacrifice, utility, and escape strategy subscales for the father–child version were 0.90, 0.81, 0.76, 0.79, and 0.81. For the mother–child version they were 0.89, 0.80, 0.76, 0.80, and 0.79, respectively.

### Analysis Strategy

As in Study 1, we followed Little et al. (2002) item-to-construct balancing procedures to parcel the RFP, AFP, and FAC items into three indicators. We did not parcel the items for the conflict resolution strategies because each has only four items. The fit indices and their acceptability criteria were the same as those in Study 1. We also analyzed the influence of parental education level as a control variable.

To detect the influence of adolescents’ gender, we adopted multi-group structural equation modeling with loading invariance for father–son and father–daughter datasets, and for mother–son and mother–daughter datasets. According to Chen (2007), loadings can be considered invariant when a change is less than 0.010 in CFI and less than 0.015 in RMSEA. For further testing the mediation effects, we ran 5,000 bootstrapping processes as suggested by Hayes and Preacher (2010), and deemed a specific mediation effect significant if the boundary between the 125th and 4,875th (i.e., the 95% CI) excluded zero.

### Results and Discussion

The means, standard deviations, and correlation coefficients of the major variables are summarized in Table 2. For the four parent–child dyads, the pattern of correlations among RFP, AFP, and the use of different strategies was almost identical to that of Study 1. For all datasets, FAC was significantly and positively related to RFP, AFP, and the compatibility, compromise, and self-sacrifice strategies. FAC had a significant and negative correlation with the escape strategy, except for the father–son dataset. It had a significant and positive correlation with the utility strategy only for the father–son dataset.

**TABLE 2:** Correlations, means, and standard deviations of the main variables in Study 2.

	***Correlation coefficients***								***Male***		***Female***	
	***1***	***2***	***3***	***4***	***5***	***6***	***7***	***8***	***M***	***SD***	***M***	***SD***
**Father–child dataset**												
1. Reciprocal filial piety (T1)		0.58**	0.39**	0.41**	0.29**	0.16**	−0.15**	−0.26**	4.97	0.83	5.13	0.84
2. Authoritarian filial piety (T1)	0.44**		0.18**	0.27**	0.22**	0.33**	−0.21**	−0.26**	3.25	0.86	3.31	0.94
3. Function-oriented appraisal (T1)	0.40**	0.17**		0.48**	0.40**	0.19**	−0.01	−0.19**	2.77	0.99	2.60	0.96
4. Compatibility strategy (T2)	0.45**	0.23**	0.44**		0.80**	0.38**	0.05	−0.32**	2.99	0.94	2.82	0.99
5. Compromise strategy (T2)	0.34**	0.21**	0.40**	0.77**		0.45**	0.18**	−0.20**	2.63	0.81	2.52	0.87
6. Self-sacrifice strategy (T2)	0.21**	0.28**	0.14**	0.34**	0.36**		0.13**	0.06	2.12	0.67	1.86	0.68
7. Utility strategy (T2)	−0.11*	−0.12*	0.13*	0.12*	0.26**	0.18**		0.40**	2.43	0.76	2.29	0.83
8. Escape strategy (T2)	−0.25**	−0.18**	−0.07	−0.38**	−0.21**	0.15**	0.32**		2.24	0.90	2.28	0.96
**Mother–child dataset**												
1. Reciprocal filial piety (T1)		0.49**	0.44**	0.38**	0.24**	0.13**	−0.07	−0.18**	5.08	0.80	5.34	0.72
2. Authoritarian filial piety (T1)	0.43**		0.22**	0.22**	0.13**	0.30**	−0.16**	−0.15**	3.36	0.86	3.53	0.90
3. Function-oriented appraisal (T1)	0.35**	0.15**		0.46**	0.33**	0.15**	0.06	−0.16**	2.89	0.96	2.78	0.96
4. Compatibility strategy (T2)	0.40**	0.18**	0.42**		0.76**	0.33**	0.07	−0.24**	3.10	0.91	2.93	0.92
5. Compromise strategy (T2)	0.22**	0.14**	0.37**	0.76**		0.40**	0.21**	−0.10*	2.75	0.79	2.62	0.83
6. Self-sacrifice strategy (T2)	0.12*	0.32**	0.13**	0.36**	0.32**		0.07	0.18**	2.13	0.70	1.96	0.69
7. Utility strategy (T2)	−0.09	−0.08	0.08	0.14**	0.26**	0.13**		0.36**	2.46	0.82	2.34	0.81
8. Escape strategy (T2)	−0.21**	−0.07	−0.21**	−0.36**	−0.19**	0.13*	0.27**		2.17	0.85	2.25	0.91

*For both father– and mother–child datasets, in the matrix of correlation coefficients the numbers below the diagonal are from the male participants (in father–child dataset: *n* = 375; in mother–child dataset: *n* = 407); those above are from the female participants (in father–child dataset: *n* = 569; in mother–child dataset: *n* = 610).*

*T1, data collected at Time 1; T2, data collected at Time 2 after a 1-year lag.*

The degree of freedom in father–child dataset is 943, while that in mother–child dataset is 1016.

*p < 0.05, **p < 0.01.

**FIGURE 2:**
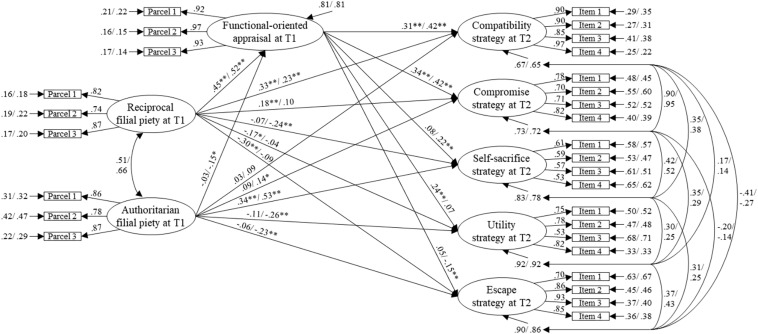
Structural model of dual filial piety, function-oriented appraisal, and parent–adolescent conflict resolution strategy for father–son and father–daughter dataset in Study 2. The influence of the parental education level has been controlled in this model. All coefficients are standardized solutions; the first coefficient is from father–son dataset (*n* = 375), the second is from father–daughter dataset (*n* = 569). T1, data collected at Time 1; T2, data collected at Time 2 after a 1-year lag. *p < 0.05, **p < 0.01.

**FIGURE 3:**
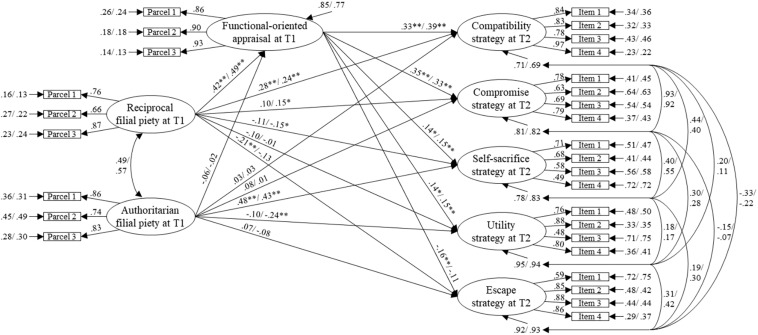
Structural model of dual filial piety, function-oriented appraisal, and parent–adolescent conflict resolution strategy for mother–son and mother–daughter dataset in Study 2. The influence of the parental education level has been controlled in this model. All coefficients are standardized solutions; the first coefficient is from mother–son dataset (*n* = 407), the second is from mother–daughter dataset (*n* = 610). T1, data collected at Time 1; T2, data collected at Time 2 after a 1-year lag. *p < 0.05, **p < 0.01.

We then investigated the mechanism linking the dual filial beliefs to different conflict resolution strategies by hypothesizing adolescents’ FAC as a mediator variable. For the father–child model (see Figure 2), the model-fit was acceptable with χ^2^(769, *N* = 944) = 1874.74, *p* < 0.001, χ^2^/*df* = 2.44, CFI = 0.93, TLI = 0.92, RMSEA = 0.055, SRMR = 0.056. All loadings were significant (*p*s < 0.001) and could be considered invariant. For the mother–child model (see Figure 3), the model-fit was also acceptable with χ^2^(769, *N* = 1017) = 1968.57, *p* < 0.001, χ^2^/*df* = 2.56, CFI = 0.93, TLI = 0.92, RMSEA = 0.055, SRMR = 0.060. All loadings were significant (*p*s < 0.001) and could be considered invariant.

The total RFP effect on the compatibility strategy for the father–son (estimate = 0.46, SE = 0.05), father–daughter (estimate = 0.45, SE = 0.06), mother–son (estimate = 0.42, SE = 0.05), and mother–daughter datasets (estimate = 0.43, SE = 0.05) was positively significant (*p*s < 0.001), as it was on the compromise strategy (in order: estimate = 0.33, SE = 0.06; estimate = 0.33, SE = 0.07; estimate = 0.25, SE = 0.06; estimate = 0.31, SE = 0.06; *p*s < 0.001). However, the total AFP effect was not significant on either the compatibility or compromise strategy for all dyad datasets. For the self-sacrifice strategy, the total AFP effect for father–son (estimate = 0.33, SE = 0.07), father–daughter (estimate = 0.50, SE = 0.06), mother–son (estimate = 0.47, SE = 0.06), and mother–daughter dataset (estimate = 0.43, SE = 0.06) was positively significant (*p*s < 0.001), but the RFP effect was not significant for any of the dyad datasets.

For the utility strategy, the total AFP effect on father– and mother–child interaction was negatively significant (*p*s < 0.001) only for the female participants (father–daughter: estimate = −0.26, SE = 0.07; mother–daughter: estimate = −0.24, SE = 0.06), and not for the male participants. The total RFP effect was not significant for any of the dyads. For the escape strategy, the RFP effect was negatively significant for the father–son (estimate = −0.28, SE = 0.06, *p* < 0.001), father–daughter (estimate = −0.17, SE = 0.07, *p* = 0.01), mother–son (estimate = −0.27, SE = 0.06, *p* < 0.001) and mother–daughter datasets (estimate = −0.19, SE = 0.06, *p* < 0.01), but the AFP effect was only negatively significant for the father–daughter dataset (estimate = −0.21, SE = 0.07, *p* < 0.01) and not for the other dyads.

The RFP effect on FAC for the father–son (γ = 0.45, SE = 0.06), father–daughter (γ = 0.52, SE = 0.06), mother–son (γ = 0.42, SE = 0.06), and mother–daughter datasets (γ = 0.49, SE = 0.04) was positively significant (*p*s < 0.001). The effect of AFP was only negatively significant for the father–daughter (γ = −0.15, SE = 0.07, *p* = 0.03) but not for the other three dyads. For all dyads, the effect of FAC was positively significant (*p*s < 0.001) on compatibility (father–son: β = 0.31, SE = 0.06; father–daughter: β = 0.42, SE = 0.04; mother–son: β = 0.33, SE = 0.05; mother–daughter: β = 0.39, SE = 0.04) and compromise (father–son: β = 0.34, SE = 0.06; father–daughter: β = 0.42, SE = 0.05; mother–son: β = 0.35, SE = 0.06; mother–daughter: β = 0.33, SE = 0.05). The effect of FAC on self-sacrifice was positively significant for father–daughter (β = 0.22, SE = 0.05, *p* < 0.001), mother–son (β = 0.14, SE = 0.06, *p* = 0.02), and mother–daughter (β = 0.15, SE = 0.06, *p* < 0.01), but not for the father–son dataset. The effect of FAC on utility was positively significant for father–son (β = 0.24, SE = 0.07, *p* < 0.001), mother–son (β = 0.14, SE = 0.06, *p* = 0.02), and mother–daughter (β = 0.15, SE = 0.06, *p* < 0.01), but not for the father–daughter dataset. The effect of FAC on escape was negatively significant for father–daughter (β = −0.15, SE = 0.06, *p* < 0.01) and mother–son (β = −0.16, SE = 0.06, *p* = 0.01), but not for the father–son and mother–daughter dataset.

We summarize the mediation effects in Table 3. For all parent–child dyad datasets, FAC significantly positively mediated the effect of RFP on the compatibility and compromise strategies. Except for the father–son dataset, the mediation effect of FAC between RFP and self-sacrifice for the father–daughter, mother–son, and mother–daughter datasets was positively significant. The mediation effect of FAC between RFP and utility was positively significant for the father–son, mother–son, and mother–daughter datasets, except for the father–daughter model. The mediation effect of FAC between RFP and escape was positively significant for only the father–daughter and mother–son datasets. For the relationship between AFP and resolution strategies, FAC significantly mediated on only use of compatibility and compromise strategy for only father–daughter dataset.

**TABLE 3:** The summary of mediation effects through function-oriented appraisal in Study 2.

	**Father–son dataset**			**Father–daughter dataset**			**Mother–son dataset**			**Mother–daughter dataset**		
	***Estimate***	***SE***	***95% CI***	***Estimate***	***SE***	***95% CI***	***Estimate***	***SE***	***95% CI***	***Estimate***	***SE***	***95% CI***
**Mediation effect from reciprocal filial piety to**												
Compatibility strategy	0.14**	**0.03**	**[0.09, 0.21]**	0.22**	**0.03**	**[0.16, 0.29]**	0.14**	**0.03**	**[0.09, 0.21]**	0.19**	**0.03**	**[0.14, 0.25]**
Compromise strategy	0.15**	**0.03**	**[0.10, 0.22]**	0.22**	**0.04**	**[0.16, 0.29]**	0.15**	**0.03**	**[0.09, 0.22]**	0.16**	**0.03**	**[0.11, 0.22]**
Self-sacrifice strategy	0.04	0.03	[−0.02, 0.10]	0.11**	**0.03**	**[0.06, 0.19]**	0.06*	**0.03**	**[0.01, 0.12]**	0.08*	**0.03**	**[0.02, 0.14]**
Utility strategy	0.11**	**0.03**	**[0.05, 0.18]**	0.04	0.03	[−0.02, 0.10]	0.06*	**0.03**	**[0.01, 0.12]**	0.08**	**0.03**	**[0.02, 0.13]**
Escape strategy	0.02	0.03	[−0.03, 0.08]	−0.08*	**0.03**	**[−0.14, −0.02]**	−0.07*	**0.03**	**[−0.12, −0.02]**	−0.06	0.03	[−0.12, 0.00]
**Mediation effect from authoritarian filial piety to**												
Compatibility strategy	−0.01	0.03	[−0.06, 0.04]	−0.06*	**0.03**	**[−0.12, −0.01]**	−0.02	0.03	[−0.07, 0.03]	−0.01	0.02	[−0.05, 0.04]
Compromise strategy	−0.01	0.03	[−0.07, 0.04]	−0.06*	**0.03**	**[−0.13, −0.01]**	−0.02	0.03	[−0.08, 0.03]	−0.01	0.02	[−0.05, 0.03]
Self-sacrifice strategy	−0.00	0.01	[−0.04, 0.01]	−0.03	0.02	[−0.08, 0.00]	−0.01	0.01	[−0.04, 0.01]	−0.00	0.01	[−0.03, 0.02]
Utility strategy	−0.01	0.02	[−0.05, 0.03]	−0.01	0.01	[−0.04, 0.00]	−0.01	0.01	[−0.04, 0.01]	−0.00	0.01	[−0.02, 0.02]
Escape strategy	−0.00	0.01	[−0.03, 0.01]	0.02	0.01	[0.00, 0.06]	0.01	0.01	[−0.01, 0.04]	0.00	0.01	[−0.01, 0.02]

Bootstrapping sample size = 5,000. Coefficients in boldface denote significance. CI, confidence interval.

*p < 0.05, **p < 0.01.

Hypotheses 1 and 2 were again supported. Self-sacrifice strategy is an intuitive way for adolescents to perform AFP; compatibility and compromise strategies are ways to practice RFP. Hypothesis 3 was partially supported. The results showed that RFP was only negatively associated with the escape strategy for all parent–child dyads, and AFP was negatively related to utility and escape strategies for some dyads.

Hypothesis 4 was also supported. Chinese adolescents valuing RFP could apply FAC to frame conflict with parents as an opportunity to improve mutual understanding, to realize what their parents expect of them, and to share intentions they want their parents to know. The adolescents could then turn this functional appraisal into behavior by adopting a compatibility or compromise strategy. The unexpected findings, those dissimilar to Study 1, and those that differed among parent–child dyads are discussed in the following.

## General Discussion

Based on results from the two empirical studies, we conclude that obedience through self-sacrifice is not the only way that Chinese adolescents practice filial piety. They can also adopt a compatibility or compromise strategy to resolve conflict, which allows them to consider parental expectations and simultaneously pursue personal goals. In addition, according to the DFPM, use of these different resolution strategies can be attributed to the reciprocal and authoritarian aspects of adolescents’ filial beliefs.

### Resolution Strategies in Line With Filial Piety

We found that the tendency to adopt a self-sacrifice strategy in parent–child conflict was associated with AFP belief. This result is in line with previous findings that adolescents endorsing AFP emphasize the hierarchical aspects of the family (Liu, 2013; Li et al., 2014; Yeh, 2014), which can lead to some maladaptive behaviors (Yeh, 2006; Leung et al., 2010). Adolescents who self-sacrifice also have problems related to maladaptation (Wu et al., 2019). These findings give rise to a common criticism of filial piety practices: If authoritarian moralism is the essential element of filial piety that causes adolescents to self-sacrifice and that consequentially results in maladaptation, why should filial piety continue to be regarded as an important virtue in modern Chinese societies?

Our findings highlight the answer. Sole consideration of AFP does not provide the full picture of filial practices. RFP is associated with other strategies for handling conflict with parents that allow adolescents to both fulfill parental expectations and achieve personal goals. In addition, previous research has indicated that those who endorse RFP may not only maintain harmonious interaction with their parents (Li et al., 2014; Chen et al., 2016), but also acquire strong individuating autonomy (Sun et al., 2019). Existing evidence also supports the association of the compatibility and compromise strategies with better adaptation in the parent–child relationship and in personal life (Yeh and Tsao, 2014; Wu et al., 2019). These findings reveal that Chinese adolescents can apply the compatibility and compromise strategies to meet their filial obligations without enduring personal sacrifice. In this respect, as a far-reaching cultural virtue, filial piety may truly balance the human need for relatedness with the need for individuation (Tsao and Yeh, 2019).

Although the effect of AFP on the compromise strategy was significantly positive in Study 1, it was not in Study 2. A possible explanation is that the effect of AFP is more variable and affected by some situational features such as parental responses (Yeh, 2012; Merolla and Kam, 2017). The surrender of an adolescent with higher AFP may first facilitate their parents in discussing conflict in a kinder manner, which then allows both parties to compromise. However, this effect may not stable enough to last for a 1-year lag.

These findings suggest a potential application. Parent–adolescent conflict is unavoidable for most adolescents (Robin and Foster, 1989), and it can cause Chinese adolescents to struggle (Kwan, 2000). On the one hand, they hope to display their inner attributes without social constraint, but on the other, they want to practice filial piety by fulfilling parental expectations (Tsao and Yeh, 2019). Based on our findings, we suggest that practitioners such as family life educators can elaborate the difference between RFP and AFP and emphasize how RFP can guide adolescents through parent–child conflict by facilitating mutual understanding, especially for those Chinese adolescents who hold only high AFP.

### Resolution Strategies in Conflict With Filial Piety

Our results indicate that both the utility and escape strategies, which reflect lower concern for parental expectations, are negatively associated with adolescents’ endorsement of the dual aspects of filial piety. These findings correspond to the conceptualization of filial piety, and reveal some noteworthy differences between RFP and AFP as well as some differences among the four parent–child dyads.

First, AFP has a more compelling effect than RFP in preventing Chinese adolescents from adopting a utility strategy. The negative effect of RFP was only significant in Study 1. It was not stable enough to reach significance with the 1-year lag in Study 2. However, the negative effect of AFP was quite robust in both studies. Previous research has likewise found that adolescents’ perceived role constraints and awareness of the obligation to obey their parents were positively correlated only with AFP, and not with RFP (Liu, 2013; Yeh, 2014). We also found that the negative effect of AFP on the utility strategy only reached significance for female adolescents, but not male adolescents. It might be because the belief in AFP stemming from Chinese authoritarian moralism also cultivates a gender frame for male youths that as the successive family leader in the future, they should demonstrate their bravery, perseverance, and determination (Shek and Lai, 2000; Li and Lamb, 2013). Thus, male adolescents highly endorsing AFP may sometimes moderately rebel against their parents to subdue this gender frame imposed on them.

Second, we found that the effect of RFP on the escape strategy was significant for the father–child and mother–child dyads in both studies, but that of AFP was fragile and differed between Study 1 (only significant for mother–child model) and Study 2 (only significant for father–daughter model). We speculate that this unstable effect of AFP could be confounded by some situational features such as parental awareness. If parents have noticed the conflict, adolescents with higher AFP would be more likely to adopt a self-sacrifice strategy to demonstrate their obedience. If parents were unaware of the conflict, however, adolescents with higher AFP may try their best to avoid going against parental authority by using the escape strategy. In contrast, because adolescents who highly endorse RFP authentically care about their parent’s expectations, they are less likely to escape whether or not their parents know about the conflicts.

### FAC as a Mediator Between Filial Piety and Conflict Resolution Strategies

We confirmed that FAC may be the underlying mechanism linking adolescents’ RFP belief to use of the compatibility and compromise strategies. This finding echoes the conceptualization of *functional conflict* that scholars proposed as a condition in which people freely express their inner attributes (e.g., Baron, 1991). FAC is defined as a key component of functional conflict (Yeh, 2012), and its adaptive effects have been identified in both parent–child (Yeh and Tsao, 2014) and romantic relationships (Chiao et al., 2018). An additional contribution from our findings is that RFP belief may be a potential antecedent for functional conflict in parent–child relationships. It can contribute to family life educators’ efforts to promote Chinese adolescents’ ability to respond to conflict with their parents.

We also found that some unexpected mediation mechanisms resulted from some significant effects of FAC on the conflict resolution strategies, namely self-sacrifice, utility, and escape. For the self-sacrifice and utility strategies, our results showed that FAC had a positively significant effect. These findings may indicate that the self-sacrifice and utility strategies can be appropriate in some situations. In some cases, adolescents may accept the rationale of their parents’ opinion and consequentially abandon their original position. In other cases, they may attempt to stand on their original position to facilitate their parents’ better understanding of their opinions. We also found that FAC had a negatively significant effect on the escape strategy. The more adolescents believe that conflict with their parents can be functional and meaningful, the less they use the escape strategy to abandon both their personal goals and parental expectations.

It is worth emphasizing, in the father–child context, the significance of the FAC’ effect on these three strategies was eliminated by the adolescents’ gender. For the father–daughter context, the effect of FAC on the utility strategy was not significant. It might be because daughters in Chinese families are generally expected to value harmony (Shek, 2000; Chao, 2008). Especially in conflict with their fathers who assume the dominant position in the family, female adolescents with high FAC might be less likely to take the risk of breaking the relationship to resist their fathers’ authority.

For the father–son context, the effect of FAC on the self-sacrifice strategy was not significant. It might be because fathers in Chinese families have the responsibility to discipline their adolescent sons as the successive leaders (Shek and Lai, 2000; Li and Lamb, 2013), and male adolescents might face a dilemma when in conflict with their fathers. Although adopting a self-sacrifice strategy can let them avoid rebellion against their fathers’ authority, it also violates their fathers’ expectation to develop them as the successive authority figure. This dilemma might weaken the negative effect of FAC on the escape strategy because some male adolescents endorsing high FAC would perceive escape as an acceptable choice in this complex situation.

Unexpectedly, we found that AFP had significantly negative impact on FAC within father–daughter dyad, and the latter in turn on the compatibility and compromise resolution strategies. As mentioned above, Chinese female adolescents who value family harmony (Shek, 2000; Chao, 2008) would try to avoid challenging the authority of their fathers. In particular, female adolescents who identify AFP would be more worry about conflicts with their fathers and do not regard conflicts with their fathers as a kind of functional event in parent–child interaction.

### Limitations and Future Directions

Several limitations are evident. The first is common method variance bias, which could have confounded the relationship between RFP and FCA in Study 2 because they were assessed at the same time. Second, although we adopted a follow-up design in Study 2, we still do not suppose the causal relationship and directionality among the changes of these variables. Further research could adopt a more precise design, such as the panel model, to remedy this limitation. Third, although previous research has verified the disparate influences of the various resolution strategies, it would be more complete if future research could directly investigate the entire mechanism of filial belief, conflict appraisal, tendency to apply particular resolution strategies, and the different adaptations in just one model. Fourth, the understanding of the detailed mechanism linking AFP to the self-sacrifice strategy is still very limited and needs further exploration and examination.

Fifth, in Study 2 we found the missing data that only participated at Time 1 but didn’t at Time 2 had significantly lower scores on RFP, AFP, and FAC than the valid data. It could be that adolescents with lower scores on these variables may be more likely to have a poor parent–child relationship and lower willingness to continue participating in our follow-up survey. This unexpected finding highlights that our results may not be fully representative and thus requires additional research to replicate the findings in future research.

Sixth, many factors that could have affected our findings were not considered in these studies, such as parental awareness, rationale of the parental advice, and parental responsiveness. Future research could investigate whether these factors might influence our findings. In addition, the factors that determine whether adolescents who emphasize RFP tend to adopt a compatibility or compromise strategy is still an unresolved issue. It is possible that the better the adolescent’s efficacy in dealing with parent–child conflict, the more likely the adolescent is to adopt compatibility rather than compromise as a strategy.

The final limitation relates to external validity. Recent research found a within-culture difference in dual filial piety among emerging adults from Taiwan, Hong Kong, and China due to the unique societal social flux, political climate, and economic development (Yeh et al., 2013). This implies that our findings with Taiwanese adolescents may not be analogous to adolescents who grow up in other Chinese societies. Future research should investigate whether our findings are supported in different Chinese societies.

## Data Availability Statement

The raw data supporting the conclusions of this article will be made available by the authors, without undue reservation.

## Ethics Statement

The studies involving human participants were reviewed and approved from the appropriate review board of the National Taiwan University, and oral informed consent was obtained from the participants. Written informed consent to participate in this study was provided by the participants’ legal guardian/next of kin.

## Author Contributions

C-WW and K-HY substantial contributions to the conception or design of the work and analysis and interpretation of concepts, revising it critically for important intellectual content, final approval of the version to be published, agreement to be accountable for all aspects of the work in ensuring that questions related to the accuracy or integrity of any part of the work are appropriately investigated and resolved. Both authors contributed to the article and approved the submitted version.

## Conflict of Interest

The authors declare that the research was conducted in the absence of any commercial or financial relationships that could be construed as a potential conflict of interest.
